# Deep Margin Elevation: Current Concepts and Clinical Considerations: A Review

**DOI:** 10.3390/medicina58101482

**Published:** 2022-10-18

**Authors:** Majed Aldakheel, Khalid Aldosary, Shatha Alnafissah, Rahaf Alaamer, Anwar Alqahtani, Nora Almuhtab

**Affiliations:** 1 Dental Department, King Abdulaziz University Hospital, King Saud University , Riyadh 11323, Saudi Arabia; 2 BDS, College of Dentistry, Princess Nourah Bint Abdul Rahman University, Riyadh 13414, Saudi Arabia; 3 BDS, College of Dentistry, King Saud University, Riyadh 12371, Saudi Arabia

**Keywords:** deep margin elevation, cervical margin relocation, proximal box elevation, sub-gingival margins

## Abstract

Dietschi and Spreafico first proposed deep margin elevation (DME) in 1998 to address the multiple clinical problems associated with sub-gingival margins, where sub-gingival margins will be repositioned coronally using composite resin restorations. Given that dentistry is directing towards conservatism, its use is currently trending. Materials and Methods: a search was performed through PubMed, Scopus, and Google Scholar search engines to obtain relevant articles with no time restriction. Results: With biological width taken into consideration, well-defined and polished sub-gingival restorations are compatible with periodontal health. Marginal integrity in the DME technique seems to be affected by the type of adhesive, restoration, and incremental layering of the restoration. Regarding fracture resistance, DME has no significant effects. Conclusion: The DME technique seems to be a minimally invasive alternative to surgical crown lengthening (SCL) and orthodontic extrusion (OE) with respect to biological width. Well-controlled clinical trials are limited in this field; further long-term follow-up studies emphasizing the periodontal outcomes and prevention of complications are needed.

## 1. Introduction

Deep margin elevation (DME), or coronal margin relocation (CMR), is a procedure used to raise or reposition sub-gingival margins into supra-gingival margins using several materials to increase marginal integrity and bonding strength [1,2,3]. Dietschi and Spreafico proposed the DME technique in 1998 to solve the problems associated with sub-gingival restorations [1]. Despite this fact, it is still considered a new approach [4]. Nowadays, clinical dentistry is directed toward conservatism, where in several situations the minimally invasive DME can replace the invasive procedures of crown lengthening [5]. The surgical approach might be accompanied by anatomic complications, such as the proximity to root concavities, furcation area, and attachment loss [2]. Sub-gingival preparations present difficulties that may complicate all further steps, such as rubber dam isolation, impression taking both digitally and traditionally [3,4,6,7], placement of a restoration, cementation as well as cervical area finishing and polishing [6,8]. Moreover, indirect partial posterior restorations often display sub-gingival margins, which are accompanied by both biological and operative problems. Biological problems may result in gingival inflammation and biological width violation [9]. While operative problems are attributed to changes in the tooth structure that are associated with deep margins, such as the absence of enamel, where dentin and cementum will pose more difficulties in bonding [3,10]. Up until now, there is a limitation in the studies assessing the advantages and limitations of DME [11], most of them are in vitro concentrating on fracture resistance [12,13], bond strength [14], and marginal adaptation of indirect restorations [3,12,15,16]. Therefore, the primary goal of our study was to address the applicability of DME compared with crown lengthening, as well as the currently available clinical parameters related to this technique.

## 2. Materials and Methods

**Figure 1:**
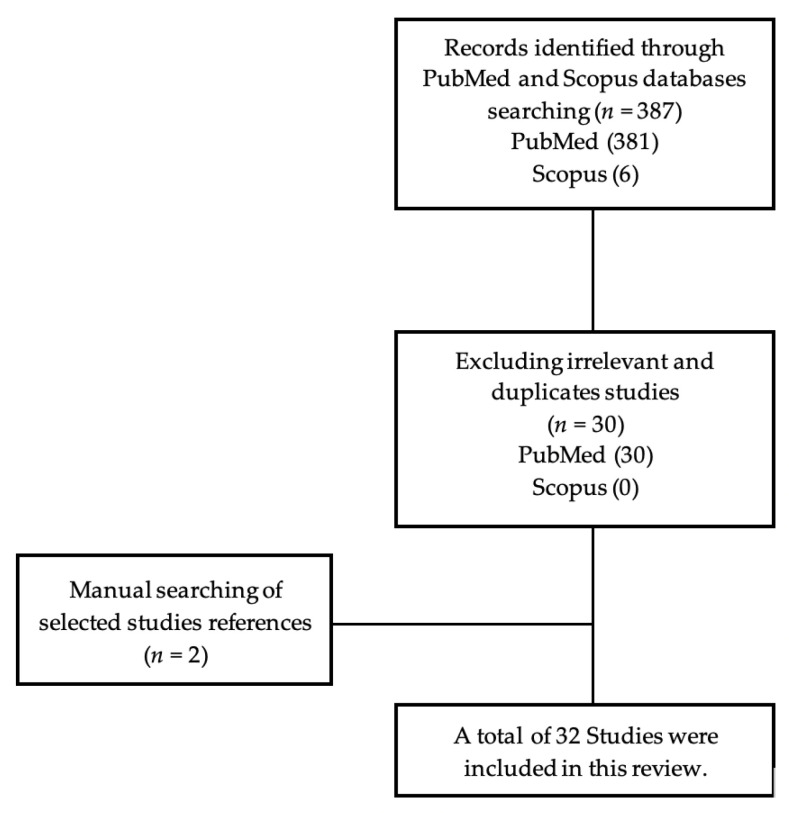
Flow chart of the studies selection.

Using a narrative search methodology carried out from October 2021 to February 2022 PubMed and Scopus databases were searched, along with the use of the Google Scholar search engine, to obtain relevant articles. Only articles published in English were included, without time restriction. The following keywords were used: deep margin elevation; cervical margin relocation; proximal box elevation; sub-gingival margins. Additional relevant articles were found by manually searching peer-reviewed journals and cross-referencing the selected articles as illustrated in Figure 1.

## 3. Results

After excluding non-relevant studies, this review included a total of 32 studies. A summary of collected evidence is presented in Table 1, which includes the study design, the investigated factors, and their main outcome.

### 3.1. Deep Margin Elevation Concept

**Figure 2:**
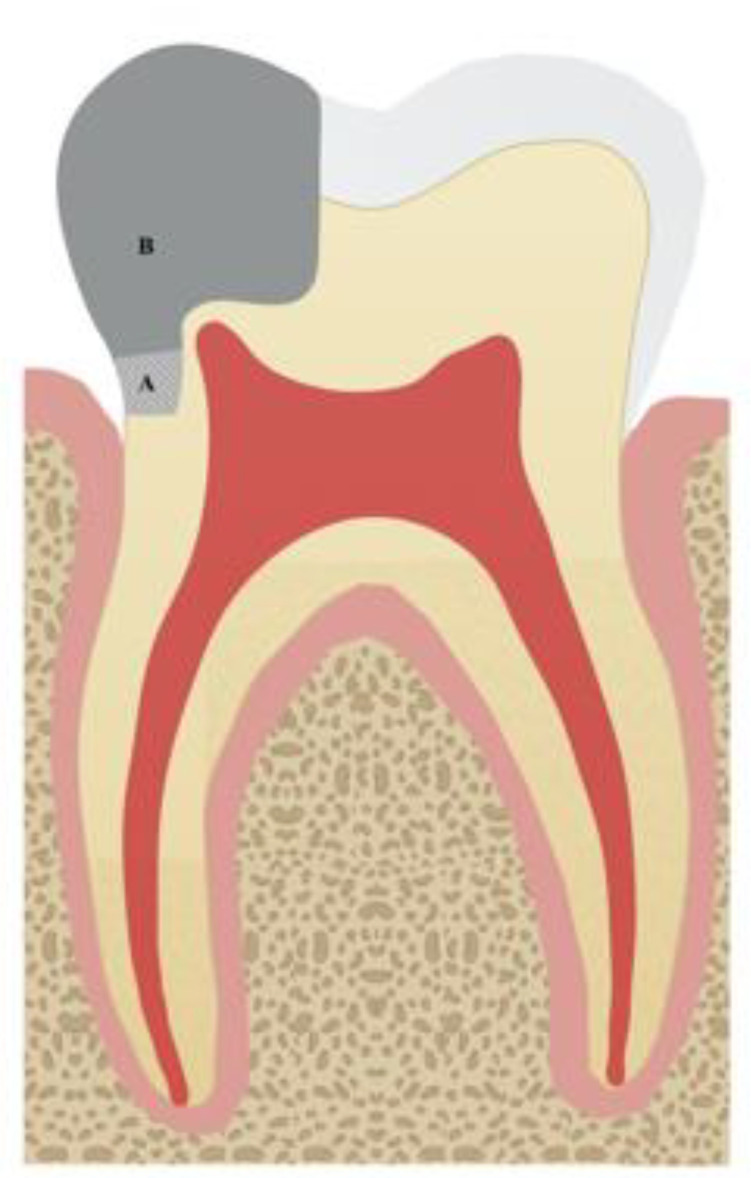
The deep margin elevation concept. (**A**) A layer of sub-gingival composite. (**B**) The final restoration.

In recent years there has been a growing interest in the field of deep margin elevation (DME) [9,12,14,15,16,17,18,19,20,21,35,36]. As a replacement for periodontal surgical procedures [21,22], a less lengthy and costly approach is DME, which presents a viable alternative to surgical crown lengthening (SCL) [13]. The DME technique was proposed back in 1998 by Dietschi et al., [1]. In the literature, there are several names given to this technique: coronal margin relocation (CMR), proximal box elevation (PBE), proximal margin elevation, cervical margin relocation, marginal elevation, and open sandwich technique [5,12,13,15,17,18,23,24,37,38,39]. When at all possible, sub-gingival margins should be avoided [40]. However, in clinical practice, extensive deep caries and sub-gingival margins are frequently encountered and impose significant challenges to the clinician [3,6,8]. Besides the technical complexity of restoring these cavities, the insufficient isolation of localized sub-gingival margins may make impression taking and cementation a problematic issue [8,15,17,18,25,26,41]. Sulcus fluid and gingival structures restrict contamination-free procedures that are necessary for durable adhesion as well as recurrent caries avoidance [15]. Further difficulties will be faced while dealing with marginal integrity [12,42], detecting and removing cement excesses in the sulci, and the possibility of biologic width violation [12]. Moreover, deep cavities with a complete absence of/or limited enamel at the margin, where dentin and occasionally cementum are frequently exposed, are more difficult to treat clinically [8,15,43,44,45,46,47]. SCL is frequently recommended for teeth with deep sub-gingival cavities to make the restoration process much easier [48]. Under optimum isolation and using a metal matrix placed inter-proximally, the margin of the cavity will be relocated to above gingival level using a direct composite resin layered meticulously illustrated in Figure 2 [2,6,8,9,11,15,16]. The placement of a composite base beneath indirect adhesive restorations provides a variety of benefits such as simplifying the reach to difficult areas, streamlining impressions, and improving marginal adaptation [6,14,18,27]. The immediate dentin sealing (IDS) technique, which is performed simultaneously with the DME, is another benefit of the DME approach, as evidence supports the application of an adhesive resin to the freshly cut dentin. Increased retention, reduced marginal leakage, enhanced bond strength, and lower postoperative sensitivity are all benefits of IDS combined with DME [49]. In addition, the extensive thickness of indirect restorations is decreased when DME is performed underneath [12,18], which will aid in better curing through indirect restorations [12,15,28]. Despite the obvious advantages of this technique, there is a limitation in the studies that evaluates the advantages and disadvantages of DME. What we know about DME is largely based on in vitro studies and case reports.

### 3.2. Periodontal Aspects

The periodontium serves as the foundation for any dental field, especially restorative dentistry [50]. Maintaining a healthy periodontium around sub-gingivally restored teeth necessitates the presence of ideal restoration that is contoured correctly [18,21,50,51]. Although supragingival margins are preferred by clinicians to maintain a healthy periodontium, many clinical scenarios, such as pre-existing deep margins, esthetic demands, or the need for retention form, might necessitate sub-gingival margins [51]. Predicting the response of gingival tissues to sub-gingival restorations depends on several aspects such as the contour of restorations and their margins, iatrogenic factors represented by overhangs, marginal discrepancies, and the type of restorative material [52,53,54,55]. A few studies reported that bleeding on probing, recession, and attachment loss are more common with sub-gingival restorations when compared with supra-gingival restorations [52,53,54,56]. Moreover, it has been revealed in several investigations that sub-gingival restorations enhance biofilm accumulation [57,58]. The mechanical properties of composite restoration will be easily affected if air is trapped during placement [59], as increased porosity, which is critical for plaque retention, is an example of the impacts [60,61,62,63,64,65]. Whenever dealing with a restorative procedure, supra-crestal tissue attachment (STA), formerly known as biologic width (BW), must be respected in all cases, as encroaching this area will most probably lead to gingival inflammation, loss of attachment, suppuration, and bleeding [5,6,22,50,66,67]. In a study done by Gargiulo et al., in 1961, the relationship of the dentogingival junction in humans was described. They reported a mean of 0.69 mm of sulcus depth that is not accounted for biological width. A mean of 0.97 mm for epithelial attachment and 1.07 mm for connective tissue attachment combined averaged 2.04 mm [68]. A substantial variation exists in the STA dimension based on periodontal health, tooth type, site, and time of healing after/prior to surgery [48]. Therefore, an attempt to measure the STA dimension for each situation is forethoughtful instead of relying on mean values. An easy and reliable method in comparison to bone sounding is trans-gingival probing [69], although probing force [70] and tissue inflammation might affect it [71]. When evaluating the effectiveness and success of the DME technique, we must assess the periodontium health in terms of bleeding on probing (BoP) and marginal bone level through radiographs [9]. Newcomb (1974) assessed the relationship between the location of sub-gingival crown margins and gingival inflammation. He reached the conclusion that the closer the margins to epithelial attachment, the more severe the gingival inflammation [72]. Martins et al., found an association between problems in new bone growth and connective tissue attachment and a noticeable inflammatory infiltrate with sub-gingival composite restorations in dogs [73]. A 12-month clinical trial on periodontal response to crowns with sub-gingival margins was conducted by Paniz at el. They reported an increase in BoP in teeth with sub-gingival margins [74]. The results are barely distinguishable from other studies [54,75,76]. On the other hand, Bertoldi et al., studied the clinical and histological reaction of periodontal tissues to sub-gingival composite resin restorations, with outcomes showing that, with respect to biological width, well-defined sub-gingival composite restorations are compatible with gingival health, with an inflammatory infiltrate similar to the untreated natural root surface [29]. A minimum of 3 mm between the restoration margin and the bone crest was recommended by several authors to promote gingival health [50,66,67]. Valderhaug et al., measured the mean of periodontal attachment loss in 329 crowns, most of them presented with a sub-gingival margin. Crowns with sub-gingival margins were associated with a higher mean of attachment loss (1.2 mm) compared with (0.6) mm in crowns with supra-gingival margins [77]. Moreover, in 1986 Parma-Benfenati conducted an experiment on beagle dogs, assessing periodontium nature with sub-gingival margins. A 5 mm of bone loss was noted in teeth with sub-gingival margins placed at the alveolar crest [50]. A 26-year long-term clinical study found that it is injurious to have a restorative procedure with sub-gingival margins as they resulted in substantial attachment loss discovered after 1 to 3 years [54]. Nevertheless, these negative outcomes were specifically related to baseline patients with a higher number of caries related to higher plaque retention [54]. The results from a randomized clinical trial investigating the periodontal health influence of the DME pretreatment on posterior teeth restored with indirect restorations showed that inflammation of periodontal tissue had a higher prevalence in teeth that underwent DME pretreatment than teeth without DME at a 1-year follow-up [9]. Another clinical trial reported the association between DME and increased bleeding on probing (BoP), which remarkably indicates compromised periodontal health, which points to the importance of the distance between the alveolar crest and the restorative margins [78]. Upon the sub-gingival placement of composite, different patterns of supra-crestal attachment were observed. It is important to note that the long junctional epithelium is the only mean available to achieve periodontal attachment to the material, as when examined histologically, it was obvious that no connective attachment could be obtained on the material [5].

### 3.3. DME versus Surgical Crown Lengthening

Sub-gingival margins are frequently encountered in clinical practice and it is critical to maintain supra-crestal tissue attachment while restoring them [8]. In such circumstances, SCL is frequently recommended to maintain a healthy periodontium [27]. Crown lengthening is indicated whenever the distance between the margin of restoration and the alveolar crest is equal to or less than 3 mm [50]. In SCL, the cavity margins will be relocated supragingivally by displacing the periodontal attachment apically [8]. It is debatable whether SCL re-creates biological width or produces gingival rebound [79]. Multiple approaches for SCL are available, including gingivectomy and apically positioned flap (APF) with or without bone resection [58]. The gingivectomy approach is associated with less postoperative morbidity compared with flap surgery [80] and it is indicated in the case of sufficient width of keratinized tissues (≥3 mm) [81] and no violation of STA [50]. APF is indicated in the case of insufficient width of keratinized tissues (<3 mm) [81] and/or osseous resection essential for re-establishing STA apico-coronal dimension [50]. To provide adequate distance from the alveolar crest to the margin of restoration, bone reduction is often mandatory [50]. A period of time must be given after crown lengthening for periodontal tissues to heal and stabilize. Five to six months were recommended by Veneziani et al., for restorations placed in esthetic zones [8]. Despite the advantages of crown lengthening, estimating the final position of margins is difficult, as mentioned by Pilalas in 2016 [82]. SCL with osseous resection may increase the risk of extraction of endodontically treated posterior teeth more than twofold after ten years due to the deleterious effect of the crown-to-root ratio [83]. The endodontic treatment outcome could be jeopardized, as prolonged healing time could delay the provision of definitive restoration [84]. Long-term clinical studies showed that after 10–13 years, more or less half of endodontically treated teeth with SCL and osseous resection will be lost [82,85]. Other disadvantages are opening the proximal contact [86] and exposure of furcation [2], which both might result in more complicated oral hygiene [2,86].

An alternative that is claimed to be less invasive is DME, which uses a direct composite restoration to raise the gingival margin into supra-gingival levels [1,17,18,30,87,88]. This can be done at a 2 mm distance from the alveolar crest with composite (considering good adaptation and polishing of the composite) as the space is preserved for the connective tissue attachment. On the contrary, lower distance is an indication of SCL with ostectomy to provide this space for the connective tissue [89]. Three different clinical situations were classified by Veneziani [8] based on technical operating and biological parameters illustrated in Table 2.

Only In grade I, when it is applicable to apply rubber dam correctly in the sulcus to show the cervical margin, DME can be performed. Other clinical situations demand surgical exposition of the margin in grade II or SCL in grade III for isolation of the operating field [8]. The debate is ongoing on whether it is better to elevate the margin non-invasively or to perform SCL to facilitate the placement of large direct composite restorations. Despite the recommendations for a conservative approach, it fails in situations where a change in the shape of the tissues is needed around the tooth for restoration [5]. Treatment choice might also be affected by furcation, root concavity, and medical history [31]. One of the most critical parts of DME outcome success is determining whether periodontal healing will occur around sub-gingival restorations; it has been hypothesized that the outcome is strongly influenced by the gingival biotype [5]. As reported by Stetler et al., a higher gingival index was associated with sub-gingival restorations placed on teeth with less than 2 mm of keratinized tissues [90]. A randomized controlled trial was carried out to compare the results of SCL with the DME technique. After 6 months of the trial, it has been noted that the surgery group scored higher attachment loss. However, in terms of BoP, plaque index, and pocket depth, no differences were noted between both groups [91]. In a systematic review that focused on the prognosis of SCL versus DME on severely decayed teeth [32], concerning SCL, the crown length was increased; however, it was remarkably decreased in the follow-up [92,93]. Lanning et al., found no significant changes in gingival margin position over 6 months [30]. Nevertheless, Pontoriero et al., observed remarkable changes in the gingival margin over 12 months of follow-up [94]. Distinct healing response through different biotypes and sites (buccal/lingual/interproximal) could be a possible explanation for this [95]. Compared with SCL, DME, along with indirect restorations, has a better survival rate. In addition, restorations on non-vital teeth as well as composite resin indirect restorations demonstrate survivability with DME [32]. A current case report assessed SCL vs. DME and recommended DME for deep cavities as a better alternative to SCL [1]. However, this conclusion is solely based on the biological width outcome, not on the successful retention or the survival rate [32].

### 3.4. Orthodontic Extrusion

Orthodontic extrusion (OE) is a low-magnitude force that causes coronal movement of the tooth, soft tissues, and supporting bone [96]. Moreover, a less coronal movement of tissues is a consequence of rapid extrusion [97], which is correlated with a higher incidence of root resorption [98] and ankylosis [99]. When compared with SCL, OE should be first considered by the clinician if applicable, as it might lead to poor aesthetic outcomes if it was not taken into consideration; these unaesthetic outcomes include poor crown to root ratio, gingival recession, and loss of adjacent teeth alveolar support [78]. In the esthetic zone, OE is often preferred over SCL as it precludes the need for bone removal and preserves the periodontal architecture and root contours [100,101]. Nevertheless, it is particularly indicated with medically compromised patients where surgical approaches are prohibited [102]. In 1973, Brown used vital staining techniques to investigate the effects of orthodontic tooth movement on periodontal bony defects in humans. He reported that there is a potential for a reduction of pocket depth, an increase in the attachment apparatus, and a change in the architecture of both hard and soft tissues of the periodontium [103]. Several situations hinder the use of OE, such as in the case of ankylosis, hypercementosis, furcation involvement, and short roots [104]. Other drawbacks include longer treatment duration, impaired oral hygiene, higher cost, and higher chances of relapse [104,105].

### 3.5. DME Technique

For successful DME, the next steps are recommended:

A curved matrix is preferred (greater curve or equivalent “banana band”) over a traditional matrix that allows isolation and elevation too but may result in an insufficient gingival emergence profile and contour for margins positioned in the region of the CEJ. The matrix must be supported by sufficient buccal and lingual walls, otherwise it will prevent extended elevation in buccal and lingual directions. The height of the matrix is reduced by 2 to 3 mm, because the thin matrix will glide sub-gingivally and seal the edge more effectively. The margin should be sealed by the matrix without any gingival tissue or rubber dam entrapped in between. In the case of a deep lesion, the matrix-in-a-matrix approach is achieved by sliding a sectioned fragment of metal matrix between the margin and the existing matrix [2]. Deep carious tissue is often kept to aid in the installation of the matrix, which can be removed afterward using ultrasonic tips with smooth distal and coarse mesial surfaces placed between the cavity margins and the matrix [5]. When possible, DME should be performed before endodontic treatment to benefit from improved isolation during root canal therapy [2]. If the tooth has already been treated, the success of root canal therapy should be verified and a glass-ionomer barrier should be used to seal the access to the canals during the elevation process. Finishing the margin before bonding with a fine diamond bur or oscillating tips (e.g., Hemisphere or Prep Ceram tips, KaVo) sprayed generously with water will guarantee the clearance of any debris or other dentin contamination that may have accumulated during matrix insertion. Then, immediate dentin sealing is recommended to be applied to the preparation using a three-step ERA, followed by a composite resin base application that will elevate the margin by about 2 mm. A packable or flowable composite can be used. When using a micro-hybrid or nanohybrid restorative material, preheating the material is recommended to simplify the application and to reduce the formation of interlayer gaps. A glycerin gel coat is recommended for final polymerization [2]. Once the margin has been raised, finishing is accomplished by using polishing strips and flexible disks [6]. To remove composite resin flashes a no. 12 blade or a sickle scaler can be used, and interdental flossing is also performed to check for overhangs. Before proceeding to the final preparation and impressions, a bitewing radiograph should be taken to check the presence of overhangs or gaps. To assess soft tissue health and the possible need for surgical intervention, careful follow-up is also required [2].

### 3.6. Marginal Integrity

Several clinical trials involving resin composite inlays have been reported over the last 20 years [106,107,108,109,110,111]. Indirect composite restorations might be preferred over direct restorations due to the less polymerization shrinkage involved [108,110,111]. The success of indirect partial restorations is contingent on a solid marginal seal [112,113]. Multiple in vitro studies have been carried out, where thermal and/or mechanical occlusal stresses were used to assess marginal integrity. The main findings revealed that the external margins were exceptionally good under the scanning electron microscope, but that the quality of the margins showed a substantial reduction in integrity after thermal and mechanical stresses [16,17,18]. L’ Flores et al., reported a low efficiency of scanning electron microscope (SEM) at a low magnification in detecting the marginal seal, as the leakage is not necessarily associated with a visible gap. The use of micro-computerized tomography or cutting the samples before scanning provided better detection [114]. An in vitro study has been conducted by Frankenberger et al., (2012) to test the effect of DME on the marginal integrity of resin composite inlays. Teeth were either left as controlled cases with margins extending till cemento-enamel junction (CEJ) or received DME that was applied as either one or three layers with multiple composite restorative materials. Inlays were luted to the sample. Before and after thermomechanical loading, SEM was used to assess the marginal integrity. It has been shown that bonding inlays to dentin on unraised margins scored greater gap-free margins, while applying DME in multiple layers was better in comparison to one-layer DME. Universal one-step adhesives were associated with higher gaps in dentin [15]. On the other hand, Da Silva. et al., reported that in cases where cavity margins are on dentin, universal adhesives achieved better sealing ability compared to etch and rinse adhesives (ERA). Superior sealing was noted when the margins were placed on enamel regardless of the type of adhesives used (universal or ERA) [11]. In addition, an in vitro study by Ilgenstein et al., reported no effect of DME on marginal quality or fracture integrity of endodontically treated molars restored with ceramic or composite onlays [12]. DME was discovered to increase the marginal and structural integrity of CAD/CAM ceramic inlays [33]. It was found by Dietschi et al., that the presence of a base with intermediate elastic modulus such as flowable composites produced better internal adaptation when compared with more rigid materials [26]. A flowable composite acts as a stress-absorbing layer beneath the filled hybrid composite resin restoration [6]. This could be justified by the idea of an “elastic wall”, which is based on the low modulus of elasticity and the high wettability of flowable materials, where the application of flowable materials will act as an intermediate layer [115,116]. This layer might not solely absorb the stress accompanied with polymerization shrinkage, but it absorbs the stress during functional loading as well. The efficacy of the layer in absorbing stress is dependable on the thickness and modulus, as increasing the thickness of the layer increases the efficacy of the stress absorption [117]. On the contrary, another study revealed no substantial difference in the marginal adaptation between types of composites [36]. Zhang H et al., (2021) tested the bulk-fill SDR and traditional resin composite as new resin monomers with low polymerization shrinkage to solve the microleakage issue [34]. They reported no significant difference between them, as agreed by other studies [118,119,120,121]. This may be returned to the fact that the thermal expansion coefficient of bulk-fill SDR is similar to the tooth tissue, so after temperature circulation, the microleakage will be less in the dentin margins [5]. In addition, delayed light curing [40,122] and soft-start polymerization enhance the arrangement of dual-cured composite molecules when used as a base in DME, which leads to polymerization stress release [123].

### 3.7. Fracture Resistance of Teeth Restored Using DME

Deep caries, trauma, and endodontic treatments can all alter and lower the fracture resistance of teeth [124,125]. The fragility of root canal treated teeth could account for the structural changes of enamel and dentin that arise following endodontic treatment [126,127]. Teeth exhibiting a significant loss of structure, such as in extensive MOD cavities, are recommended to receive indirect onlays after 1.5 to 2 mm minimum cuspal reduction to enhance their fracture strength [127,128]. Few researchers have addressed the issue of fracture resistance of teeth restored using DME [12,13,26,34]. The impact of DME and the materials used on fracture resistance of teeth restored with CAD/CAM ceramic and composite onlays has been investigated by Ilgenstein et al. In their methodology they compared ceramic and composite onlays with or without DME in terms of fracture resistance. Study findings demonstrate that, regardless of the type of material, DME had no effect on fracture resistance. In addition, composite onlays were superior in terms of fracture resistance when compared with ceramic onlays [12]. These results share several similarities with the findings of Grubbs et al., (2019), where fracture resistance of margins elevated by glass ionomers, resin-modified glass ionomers, composites, or bulk-fill composites after loading showed no statistically significant difference between materials [13]. In a related study, Zhang H. et al., (2021) evaluated the effect of DME and the materials used on the fracture resistance of teeth restored by ceramic endocrowns. The fracture resistance of ceramic endocrowns was increased by DME. Moreover, there was no significant difference in the type of restorative material used to raise the margin [34]. In an investigation on the effect of DME and preparation design on the fracture resistance of CAD/CAM lithium disilicate ceramic crowns, Bresser et al., showed that DME had no significant effect on fracture resistance [25].

## 4. Conclusions

According to the results observed in this review, the DME technique seems to be a minimally invasive alternative to SCL and OE with respect to biological width in terms of time, cost, and patient comfort. However, current evidence is not enough to encourage practicing this technique with as predictable outcomes as SCL and OE until long-term clinical-based studies focused on the periodontal outcomes of teeth restored with DME, their marginal integrity, and fracture resistance are established.

## Figures and Tables

**Table 1:** Summary of reviewed studies.

Authors, Year of Publication	Type of Study	Investigated Factors	Study Design/Methodology	Summary of Findings
Dietschi et al., 1998 [1]	Review	-	Described the procedures used for adhesive cementation of different types of posterior restorations.	Application of composite layer in the base of the proximal cavity under rubber dam isolation for DME is possible.
Magne et al., 2012 [2]	Review	-	An in-depth explanation of the DME technique.	Deep margin elevation can be a less invasive alternative compared with surgical crown lengthening.
Juloski et al., 2018 [3]	Review1234567	-	Discussed the effect of DME on marginal adaptation, fracture resistance, and bond strength.	No evidence of a difference in marginal quality in raised and non-raised margins. No difference in fracture resistance values among DME groups regardless of the restoration type used. With regard to bond strength, higher results were obtained with raised margins. The current evidence is not strong enough to encourage or discourage the use of DME.
Binalrimal et al., 2021 [4]	Cross-sectional	Knowledge, attitude, and practice regarding DME among dental practitioners.	Assessed the knowledge of dental practioners regarding DME and the relation between years of experience and DME knowledge.	Adequate knowledge among practitioners was observed with regard to DME.
Sarfati et al., 2018 [5]	Review and case report	-	Compared the outcomes of SCL versus DME and the periodontal response to different restorative materials.	Similar results were seen in DME and SCL regarding BoP, with higher CAL values in SCL, suggesting the well tolerance of DME by the periodontium.
Kielbassa et al., 2015 [6]	Systematic review and case report	Fracture resistance and marginal integrity.	Evaluated in vitro studies and randomized controlled trials on DME.	Fracture resistance was improved by applying flowable composite liner in class II amalgam restorations. With regard to marginal integrity, the preferred base material is controversial.
Veneziani et al., 2010 [8]	Review	-	Discussed different treatment approaches for posterior teeth with sub-gingival margins.	A new classification was established based on operative and biological parameters for dealing with sub-gingival margins.
Ferrari et al., 2018 [9]	Controlled trial	Periodontal health	Assessed the effect of DME on periodontal health. A 12-month controlled trial was obtained to assess GI, PI, and BoP in teeth restored with DME. A total of 35 teeth were divided into two groups: (G1) DME and (G2) shoulder preparation without DME. For DME, G-Premio bond and resin composite were used. Lithium disilicate crowns were luted.	A higher incidence of BoP was noticed in teeth treated with DME when margins are closer than 2 mm to the alveolar crest. No significant difference was found in GI and PI between both groups.
Da Silva et al., 2021 [11]	In vitro	Marginal seal	Assessed the effect of margin location and adhesive strategy of DME technique on the marginal seal of resin composite inlays. MOD cavities on 12 third molars were prepared and divided into six groups based on margin location and type of adhesive used as follow: (G1) Enamel + ERA (SB1XT), (G2) Dentin + ERA (SB1XT), (G3) DME + ERA (SB1XT), (G4) Enamel + SEA with enamel selective etching Clearfil SE Bond (CSE), (G5) Dentin + SEA, (G6) DME + SEA. Resin composite inlay bonded with conventional dual-cure resin.	SEA showed better sealing ability than the ERA when DME was applied or when margins were located sub-gingivally. Hermetic seal can be achieved whenever enamel margin is present with the use of selective enamel etching, regardless of the type of adhesive.
Ilgenstein et al., 2014 [12]	In vitro	Marginal quality and fracture behavior.	Assessed the marginal quality and fracture behavior of root filled molars restored with CAD/CAM fabricated ceramic and composite onlays. A total of 48 MOD cavities with distal margins 2 mm below CEJ were prepared. Proximal box elevation to CEJ with composite resin prepared in (G1+ G2), no elevation was prepared in (G3+ G4). CAD-CAM fabricated restorations with feldspathic ceramic in (G1 + G3) and resin nano-ceramic in (G2 + G4).	Marginal integrity and fracture resistance were not affected by DME.
Grubbs et al., 2020 [13]	In vitro	Marginal quality and fracture resistance	Assessed the effect of restorative material type used in DME on the marginal quality and fracture resistance of CAD/CAM fabricated onlays. A total of 75 MOD specimens prepared by CAD-CAM divided into five groups depending on the type of material used for margin elevation: (G1) type II GI, (G2) type II RMGI, (G3) RBC, (G4) BF RBC, (G5) a control with no box elevation procedure.	Restorative materials have no effect on marginal quality nor fracture resistance.
Da Silva Gonçalves et al., 2017 [14]	In vitro	Bond strength	Assessed the effects of DME on bond strength of composite inlays. Class II cavities were prepared in 25 molars and divided into four groups: (G1) RelyX ARC, without DME, (G2) RelyX ARC with DME, (G3) G-Cem without DME, (G4) G-Cem with DME.	In the case where DME was applied and G-Cem resin cement was used, the bond strength of composite inlays was significantly increased. When DME technique was applied and RelyX ARC cement was used, the bond strength was not affected.
Roggendorf et al., 2012 [15]	In vitro	Marginal quality	40 MOD cavities were raised 3 mm with one of the following materials: (G1) G-Cem, (G2) Maxcem Elite (G3 + G4) Clearfil Majesty Posterior in one or three layers, respectively, (G5) untreated “control”, then restored with resin composite inlays.	Multi-layered DME is highly effectual in bonding indirect resin composite to deep proximal boxes. Self-adhesive cement proved unsuitable for this technique.
Spreafico et al., 2016 [16]	In vitro	Marginal quality	A total of 40 molars with standard crown preparations with non-raised distal margins located in enamel as a control group. Mesial margins were located 2 mm below CEJ and raised using: (G1) Filtek Flow Supreme XTE and LAVA ultimate, (G2) Filtek Supreme XTE and LAVA Ultimate, (G3) Filtek Flow Supreme XTE and IPS e-max, (G4) Filtek Supreme XTE and IPS e-max.1234567	DME has no effect with regard to marginal integrity.
Frankenberger et al., 2013 [17]	In vitro	Marginal quality	A total of 48 MOD cavities were raised 3 mm with one of the following materials: (G1) G-Cem, (G2) Maxcem Elite (G3 + G4) Clearfil Majesty Posterior in one or three layers respectively, (G5) untreated “control”, then restored with ceramic inlays.	DME aided the bonding of ceramics to deep cervical margins. The best marginal quality was obtained by 3 layers of DME. Self-adhesive cement proved unsuitable for this technique.
Zaruba et al., 2012 [18]	In vitro	Marginal adaptation	A total of 40 MOD cavities distributed into four groups: (G1) enamel margins, (G2-4) margins 2 mm below CEJ, (G2) one 3 mm layer DME, (G3) two 1.5 mm layers DME, (G4) left untreated. Ceramic inlays were bonded to all groups	DME does not affect the marginal integrity of ceramic inlays.
Dietschi et al., 2015 [19]	Review	-	Presented a treatment protocol for dealing with bonded inlays and onlays.	DME will ease isolation, impression taking, cementation procedures, and finishing of the margins.
Rocca et al., 2015 [20]	Review	-	Presented evidence-based concepts and procedures for bonded inlays and onlays.	With regard to DME, the minimum thickness needed for locating the margin supragingivally was 1–1.5 mm.
Müller et al., 2017 [21]	In vitro	Marginal integrity	Assessed the effect of deep margin elevation on the marginal integrity of teeth restored with adhesively bonded cerec inlays in 24 molar teeth with MOD cavities extending to CEJ. DME was prepared on one of the proximal boxes using Filtek Supreme. The sample was further divided into three groups: (G1) inlays luted using Scotchbond Universal and Rely X Ultimate, (G2) inlays luted using Monobond Plus, Syntac, and Variolink II, (G3) inlays luted using Clearfil Ceramic Primer and Panavia SA Cement.	Similar marginal integrity between teeth restored with DME and teeth with inlays directly bonded to dentin.
Köken et al., 2018 [22]	In vitro	Marginal sealing	Assessed the effect of DME using hybrid composite and flowable composite on the marginal sealing of CAD/CAM MOD overlays. MOD cavities in 39 molars with a 1 mm sub-gingival margin on the mesial side, the sample was divided into three groups: (G1) = DME using Hybrid composite (GC Essentia MD), (G2) = DME using Flowable composite (GC G-aenial Universal Flo), and (G3) = no DME was prepared.	In the DME groups (1 + 2), the marginal sealing ability of both types of composites was comparable. Bonding CAD/CAM overlays directly to dentin without DME showed better marginal sealing.
Marchesi et al., 2014 [23]	In vitro	Marginal integrity	Assessed the effect of DME using Optibond FL and Filtek Supreme XTE flow on the marginal integrity of CAD/CAM fabricated lithium disilicate crowns.	DME does not affect marginal integrity.
Lefever et al., 2012 [24]	In vitro	Marginal adaptation	A total of 88 molars with a standardized box cavity where margins are located below CEJ were distributed into 11 groups according to the type of restorative materials and adhesive systems used as follows: (G1) Filtek Silorane, (G2) Clearfil AP-X, (G3) Clearfil Majesty Posterior, (G4) Filtek Silorane, (G5) Filtek Silorane, (G6) Clearfil AP-X, (G7) Clearfil Majesty Posterior, (G8) Vertise Flow, (G9) Clearfil Majesty Flow, (G10) RelyX Unicem, (G11) SDR. The adhesive systems used were: (G1–G3) Filtek Silorane Primer + bond, (G4–G7 and G9 + G11) Clearfil Protect Bond, and (G8 + G10) no adhesive was used.	Using different materials for relocating sub-gingival margins can have different results in marginal integrity.
Bresser et al., 2020 [25]	In vitro	Fracture strength	Assessed the effect of DME and preparation geometry on the fracture strength of CAD/CAM fabricated lithium disilicate restorations. A total of 60 extracted molars were randomly assigned to one of four groups: (G1) inlay without DME, (G2) inlay with DME, (G3) onlay without DME, and (G4) onlay with DME. Aging and occlusal stressing were applied to all samples.	DME did not affect the fracture strength of lithium disilicate restorations, while cuspal coverage increased the fracture strength.
Dietschi et al., 2003 [26]	In vitro	Marginal and internal adaptation.	Compared the marginal and internal adaptation of fine hybrid composite onlays with and without DME after occlusal stressing. A total of 40 molars were prepared with proximal boxes extending into the cervical margins. The type of restorative materials used for bases was as follows: Revolution (Kerr), Tetric flow (Vivadent), Dyract (Detery-Dentsply), and Prodigy (Kerr).	The bonding of inlays can be influenced by the physical properties of materials, and flowable composites can be used for relocating the margins.
Juloski et al., 2020 [27]	In vitro	Marginal quality	Assessed the marginal quality of 14 molars prepared for MOD cavities with proximal margins located in dentin. All the mesial proximal boxes were elevated and further divided into two groups depending on the type of material used: (G1) TEA and flowable composite, (G2) UA and bulk-fill flowable composite. The distal proximal boxes were not elevated.	Placing the restoration directly to dentin without DME showed better marginal sealing. In addition, the type of restorative material used affects the marginal sealing.
Alhassan et al., 2020 [28]	Case report	-	Reported a case with deep proximal cavities treated using DME.	Whenever adequate isolation and feasible matrix placement can be achieved, DME can be used.
Bertoldi et al., 2019 [29]	Clinical study	Clinical and histological response.	Assessed the response of periodontal tissues to sub-gingival restorations when compared with untreated root surfaces. DME was applied on 29 teeth with sub-gingival cavities.	With respect to biological width and following a firm supportive therapy, DME is compatible with periodontal tissues.
Frese et al., 2014 [30]	Review and case report	-	Presented a step-by-step technique for DME in a case where biological width was invaded.	The 12-month follow-up period showed no signs of hard or soft tissue inflammation.
Dablanca-Blanco et al., 2017 [31]	Case reports	-	Discussed seven different scenarios of molars with deep sub-gingival margins, their treatment approaches, and the indication for DME vs. SCL.	Whenever optimal matrix placement can be achieved, the DME technique can be used. Otherwise, in deeper cavities that invades the BW, SCL is recommended.
Mugri et al., 2021 [32]	Systematic review	Survival rate	Compared the survival rate of teeth restored with SCL versus DME.	A higher long-term predictability of teeth treated using SCL was noticed. However, the survival ratio of DME treated teeth was higher than SCL.
Vertolli et al., 2020 [33]	In vitro	Structural and marginal integrity	Assessed the effects of DME on the structural and marginal integrity of teeth restored using CAD/CAM fabricated ceramic inlays. A total of 40 molars were separated into four groups as follows: (G1) enamel margins, (G2) margins 2 mm below CEJ, (G3) margins 2 mm below CEJ and elevated with GIC, (G4) margins 2 mm below CEJ and elevated with RMGI. The class II inlays were bonded to all teeth.	Margins placed in cementum had a higher ceramic fracture rate and DME was not affected by the type of restoration GI or RMGI.
Zhang et al., 2021 [34]	In vitro	Fracture resistance and microleakage	Assessed the effects of DME on the fracture resistance and microleakage of teeth restored using ceramic endocrowns.	Fracture resistance in raised margins had higher values in comparison with non-raised margins.

Abbreviations: SCL—surgical crown lengthening; BoP—bleeding on probing; CAL—clinical attachment loss; GI—gingival index; PI—plaque index; MOD—mesio occluso distal; ERA—etch and rinse adhesive; SEA—self-etching adhesive; CEJ—cemento enamel junction; GI—glass ionomer; RMGI—resin modified glass ionomer; RBC—resin-based composite; BF—bulk-fill resin-based composite. TEA—total etch adhesive; UA—universal adhesive.

**Table 2:** Classification of adhesive restorations with sub-gingival margins based on technical operating and biological parameters.

**Grade I**	On placement of rubber dam in the gingival sulcus, the cervical margin can be adequately visible.
**Grade II**	A rubber dam is not sufficient to isolate the field, yet biological width is respected.
**Grade III**	Deep sub-gingival margins violating the biological width.

## Data Availability

Not applicable.

## References

[1] Dietschi D., Spreafico R. (1998). Current Clinical Concepts for Adhesive Cementation of Tooth-Colored Posterior Restorations. Pract. Periodont. Aesthet. Dent..

[2] Magne P., Spreafico R.C. (2012). Deep Margin Elevation: A Paradigm Shift. Am. J. Esthet. Dent..

[3] Juloski J., Köken S., Ferrari M. (2018). Cervical Margin Relocation in Indirect Adhesive Restorations: A Literature Review. J. Prosthodont. Res..

[4] Binalrimal S.R., Banjar W.M., Alyousef S., Alawad M., Alawad G. (2021). Assessment of Knowledge, Attitude, and Practice Regarding Deep Margin Elevation (DME) Among Dental Practitioners in Riyadh, Saudi Arabia. Fam. Med. Prim. Care Rev..

[5] Sarfati A., Tirlet G. (2018). Deep Margin Elevation Versus Crown Lengthening: Biologic Width Revisited. Int. J. Esthet. Dent..

[6] Kielbassa A.M., Philipp F. (2015). Restoring Proximal Cavities of Molars Using the Proximal Box Elevation Technique: Systematic Review and Report of a Case. Quintessence Int..

[7] D’Arcangelo C., Vanini L., Casinelli M., Frascaria M., De Angelis F., Vadini M., D’Amario M. (2015). Adhesive Cementation of Indirect Composite Inlays and Onlays: A Literature Review. Compend. Contin. Educ. Dent..

[8] Veneziani M. (2010). Adhesive Restorations in the Posterior Area with Subgingival Cervical Margins: New Classification and Differentiated Treatment Approach. Eur. J. Esthet. Dent..

[9] Ferrari M., Koken S., Grandini S., Ferrari Cagidiaco E., Joda T., Discepoli N. (2018). Influence of Cervical Margin Relocation (CMR) on Periodontal Health: 12-Month Results of a Controlled Trial. J. Dent..

[10] Cardoso M., de Almeida Neves A., Mine A., Coutinho E., Van Landuyt K., De Munck J., Van Meerbeek B. (2011). Current Aspects on Bonding Effectiveness and Stability in Adhesive Dentistry. Aust. Dent. J..

[11] Da Silva D., Ceballos L., Fuentes M. (2021). Influence of the Adhesive Strategy in the Sealing Ability of Resin Composite Inlays After Deep Margin Elevation. J. Clin. Exp. Dent..

[12] Ilgenstein I., Zitzmann N., Bühler J., Wegehaupt F., Attin T., Weiger R., Krastl G. (2014). Influence of Proximal Box Elevation on the Marginal Quality and Fracture Behavior of Root-Filled Molars Restored with CAD/CAM Ceramic or Composite Onlays. Clin. Oral. Investig..

[13] Grubbs T., Vargas M., Kolker J., Teixeira E. (2020). Efficacy of Direct Restorative Materials in Proximal Box Elevation on the Margin Quality and Fracture Resistance of Molars Restored with CAD/CAM Onlays. Oper. Dent..

[14] Da Silva Gonçalves D., Cura M., Ceballos L., Fuentes M. (2017). Influence of Proximal Box Elevation on Bond Strength of Composite Inlays. Clin. Oral. Investig..

[15] Roggendorf M., Krämer N., Dippold C., Vosen V., Naumann M., Jablonski-Momeni A., Frankenberger R. (2012). Effect of Proximal Box Elevation with Resin Composite on Marginal Quality of Resin Composite Inlays in Vitro. J. Dent..

[16] Spreafico R., Marchesi G., Turco G., Frassetto A., Di Lenarda R., Mazzoni A., Cadenaro M., Breschi L. (2016). Evaluation of the in Vitro Effects of Cervical Marginal Relocation Using Composite Resins on the Marginal Quality of CAD/CAM Crowns. J. Adhes. Dent..

[17] Frankenberger R., Hehn J., Hajtó J., Krämer N., Naumann M., Koch A., Roggendorf M. (2013). Effect of Proximal Box Elevation with Resin Composite on Marginal Quality of Ceramic Inlays in Vitro. Clin. Oral. Investig..

[18] Zaruba M., Göhring T., Wegehaupt F., Attin T. (2012). Influence of a Proximal Margin Elevation Technique on Marginal Adaptation of Ceramic Inlays. Acta Odontol. Scand..

[19] Dietschi D., Spreafico R. (2015). Evidence-Based Concepts and Procedures for Bonded Inlays and Onlays. Part I. Historical Perspectives and Clinical Rationale for a Biosubstitutive Approach. Int. J. Esthet. Dent..

[20] Rocca G.T., Rizcalla N., Krejci I., Dietschi D. (2015). Evidence-Based Concepts and Procedures for Bonded Inlays and Onlays. Part I.I. Guidelines for Cavity Preparation and Restoration Fabrication. Int. J. Esthet. Dent..

[21] Müller V., Friedl K., Friedl K., Hahnel S., Handel G., Lang R. (2017). Influence of Proximal Box Elevation Technique on Marginal Integrity of Adhesively Luted Cerec Inlays. Clin. Oral Investig..

[22] Köken S., Juloski J., Sorrentino R., Grandini S., Ferrari M. (2018). Marginal Sealing of Relocated Cervical Margins of Mesio-Occluso-Distal Overlays. J. Oral Sci..

[23] Marchesi G., Spreafico R., Frassetto A., Turco G., Di Lenarda R., Cadenaro M., Breschi L. (2014). Cervical Margin-Relocation of CAD/CAM Lithium Disilicate Ceramic Crown Using Resin-Composite. Dent. Mater..

[24] Lefever D., Gregor L., Bortolotto T., Krejci I. (2012). Supragingival Relocation of Subgingivally Located Margins for Adhesive Inlays/Onlays with Different Materials. J. Adhes. Dent..

[25] Bresser R., van de Geer L., Gerdolle D., Schepke U., Cune M., Gresnigt M. (2020). Influence of Deep Margin Elevation and Preparation Design on the Fracture Strength of Indirectly Restored Molars. J. Mech. Behav. Biomed. Mater..

[26] Dietschi D., Olsburgh S., Krejci I., Davidson C. (2003). in vitro evaluation of marginal and internal adaptation after occlusal stressing of indirect class II composite restorations with different resinous bases. Eur. J. Oral. Sci..

[27] Juloski J., Köken S., Ferrari M. (2020). No Correlation Between Two Methodological Approaches Applied to Evaluate Cervical Margin Relocation. Dent. Mater. J..

[28] Alhassan M.A., Bajunaid S.O. (2020). Effect of cervical margin relocation technique with composite resin on the marginal integrity of a ceramic onlay: A case report. Gen. Dent..

[29] Bertoldi C., Monari E., Cortellini P., Generali L., Lucchi A., Spinato S., Zaffe D. (2019). Clinical and Histological Reaction of Periodontal Tissues to Subgingival Resin Composite Restorations. Clin. Oral Investig..

[30] Frese C., Wolff D., Staehle H.J. (2014). Proximal box elevation with resin composite and the dogma of biological width: Clinical R2-technique and critical review. Oper. Dent..

[31] Dablanca-Blanco A.B., Blanco-Carrión J., Martín-Biedma B., Varela-Patiño P., Bello-Castro A., Castelo-Baz P. (2017). Management of large class II lesions in molars: How to restore and when to perform surgical crown lengthening?. Restor. Dent. Endod..

[32] Mugri M.H., Sayed M.E., Nedumgottil B.M., Bhandi S., Raj A.T., Testarelli L., Khurshid Z., Jain S., Patil S. (2021). Treatment Prognosis of Restored Teeth with Crown Lengthening vs. Deep Margin Elevation: A Systematic Review. Materials.

[33] Vertolli T.J., Martinsen B.D., Hanson C.M., Howard R.S., Kooistra S., Ye L. (2020). Effect of Deep Margin Elevation on CAD/CAM-Fabricated Ceramic Inlays. Oper. Dent..

[34] Zhang H., Li H., Cong Q., Zhang Z., Du A., Wang Y. (2021). Effect of proximal box elevation on fracture resistance and microleakage of premolars restored with ceramic endocrowns. PLoS ONE.

[35] Zaruba M., Kasper R., Kazama R., Wegehaupt F., Ender A., Attin T., Mehl A. (2013). Marginal Adaptation of Ceramic and Composite Inlays in Minimally Invasive Mod Cavities. Clin. Oral Investig..

[36] Rocca G.T., Gregor L., Sandoval M.J., Krejci I., Dietschi D. (2012). in vitro evaluation of marginal and internal adaptation after occlusal stressing of indirect class II composite restorations with different resinous bases and interface treatments. Post-fatigue adaptation of indirect composite restorations. Clin. Oral Investig..

[37] Lindberg A., van Dijken J.W., Lindberg M. (2007). Nine-Year Evaluation of a Polyacid-Modified Resin Composite/Resin Composite Open Sandwich Technique in Class II Cavities. J. Dent..

[38] Shafiei F., Akbarian S. (2014). Microleakage of Nanofilled Resin-Modified Glass-Ionomer/Silorane- Or Methacrylate-Based Composite Sandwich Class II Restoration: Effect of Simultaneous Bonding. Oper. Dent..

[39] Atlas A.M., Raman P., Dworak M., Mante F., Blatz M.B. (2009). Effect of Delayed Light Polymerization of a Dual-Cured Composite Base on Microleakage of Class 2 Posterior Composite Open-Sandwich Restorations. Quintessence Int..

[40] Kamin S. (1989). the biologic width--periodontal-restorative relationship. Singapore Dent. J..

[41] Rochdi T., Nouha M., Hayet H., Abdellatif B. (2018). Deep margin elevation for indirect bonded restorations: A clinical report. Sch. J. Dent. Sci..

[42] Kanca III J., GREITZER G. (2009). Class II Restorations with Margins below the CE. J. Esthet. Restor. Dent..

[43] De Munck J., Van Landuyt K., Peumans M., Poitevin A., Lambrechts P., Braem M., Van Meerbeek B. (2005). a Critical Review of the Durability of Adhesion to Tooth Tissue: Methods and Results. J. Dent. Res..

[44] Cavalcanti A.N., Mitsui F.H.O., Lima A.F., Mathias P., Marchi G.M. (2010). Evaluation of Dentin Hardness and Bond Strength At Different Walls of Class II Preparations. J. Adhes. Dent..

[45] Van Meerbeek B., Peumans M., Poitevin A., Mine A., Van Ende A., Neves A., De Munck J. (2010). Relationship Between Bond-Strength Tests and Clinical Outcomes. Dent. Mater..

[46] Mormann W., Regolati B., Renggli H. (1974). Gingival Reaction to Well-Fitted Subgingival Proximal Gold Inlays. J. Clin. Periodontol..

[47] Ariaans K., Heussen N., Schiffer H., Wienert A., Plümäkers B., Rink L., Wolfart S. (2016). Use of Molecular Indicators of Inflammation to Assess the Biocompatibility of All-Ceramic Restorations. J. Clin. Periodontol..

[48] Schmidt J., Sahrmann P., Weiger R., Schmidlin P.R., Walter C. (2013). Biologic Width Dimensions—A Systematic Review. J. Clin. Periodontol..

[49] Magne P. (2014). IDS: Immediate Dentin Sealing (IDS) for tooth preparations. J. Adhes. Dent..

[50] Padbury A., Eber R., Wang H. (2003). Interactions Between the Gingiva and the Margin of Restorations. J. Clin. Periodontol..

[51] Paolantonio M., D’ercole S., Perinetti G., Tripodi D., Catamo G., Serra E., Brue C., Piccolomini R. (2004). Clinical and Microbiological Effects of Different Restorative Materials on the Periodontal Tissues Adjacent to Subgingival Class V Restorations. 1-Year Results. J. Clin. Periodontol..

[52] Waerhaug J. (1956). Effect of rough surfaces upon gingival tissue. J. Dent. Res..

[53] Larato D.C. (1972). Influence of a composite resin restoration on the gingiva. J. Prosthet. Dent..

[54] Schatzle M., Land N.P., Anerud A., Boysen H., Burgin W., Loë H. (2001). the influence of margins of restorations of the periodontal tissues over 26 years. J. Clin. Periodontol..

[55] Roman-Torres C., Cortelli S., de Araujo M., Aquino D., Cortelli J. (2006). a Short-Term Clinical and Microbial Evaluation of Periodontal Therapy Associated with Amalgam Overhang Removal. J. Periodontol..

[56] Waerhaug J. (1960). Histologic Considerations Which Govern Where the Margins of Restorations Should Be Located in Relation to the Gingiva. Dent. Clin. N. Am..

[57] Waerhaug J. (1975). Presence or Absence of Plaque on Subgingival Restorations. Eur. J. Oral Sci..

[58] Van Dijken J.W., Sjostrom S. (1991). the Effect of Glass Ionomer Cement and Composite Resin Fillings on Marginal Gingiva. J. Clin. Periodontol..

[59] Truffier-Boutry D., Gallez X., Demoustier-Champagne S., Devaux J., Mestdagh M., Champagne B., Leloup G. (2003). Identification of Free Radicals Trapped in Solid Methacrylated Resins. J. Polym. Sci. Polym. Chem..

[60] Jørgensen K.D. (1980). Restorative resins: Abrasion vs. mechanical properties. Scand. J. Dent. Res..

[61] van Dijken J.W., Ruyter I.E., Holland R.I. (1986). Porosity in posterior composite resins. Scand. J. Dent. Res..

[62] McCabe J.F., Ogden A.R. (1987). the Relationship Between Porosity, Compressive Fatigue Limit and Wear in Composite Resin Restorative Materials. Dent. Mater..

[63] Opdam N.J., Roeters J.J., Joosten M., Veeke O. (2002). Porosities and voids in class I restorations placed by six operators using a packable or syringable composite. Dent. Mater..

[64] Tantbirojn D., Versluis A., Cheng Y.S., Douglas W.H. (2003). Fracture toughness and microhardness of a composite: Do they correlate?. J. Dent..

[65] Drummond J.L. (2008). Degradation, Fatigue, and Failure of Resin Dental Composite Materials. J. Dent. Res..

[66] Planciunas L., Puriene A., Mackeviciene G. (2006). Surgical lengthening of the clinical tooth crown. Stomatologija.

[67] Mishkin D.J., Gellin R.G. (1993). Re: Biologic width and crown lengthening. J. Periodontol..

[68] Gargiulo A., Wentz F., Orban B. (1961). Dimensions and Relations of the Dentogingival Junction in Humans. J. Periodontol..

[69] Perez J.R., Smukler H., Nunn M.E. (2007). Clinical evaluation of the supraosseous gingivae before and after crown lengthening. J. Periodontol..

[70] Van der Velden U. (1979). Probing Force and the Relationship of the Probe Tip to the Periodontal Tissues. J. Clin. Periodontol..

[71] Armitage G.C. (1996). Manual periodontal probing in supportive periodontal treatment. Periodontol. 2000.

[72] Newcomb G. (1974). the Relationship Between the Location of Subgingival Crown Margins and Gingival Inflammation. J. Periodontol..

[73] Martins T., Bosco A., Nóbrega F., Nagata M., Garcia V., Fucini S. (2007). Periodontal Tissue Response to Coverage of Root Cavities Restored with Resin Materials: A Histomorphometric Study in Dogs. J. Periodontol..

[74] Paniz G., Nart J., Gobbato L., Mazzocco F., Stellini E., De Simone G., Bressan E. (2017). Clinical Periodontal Response to Anterior All-Ceramic Crowns with Either Chamfer or Feather-Edge Subgingival Tooth Preparations: Six-Month Results and Patient Perception. Int. J. Periodontics Restor. Dent..

[75] Valderhaug J., Ellingsen J., Jokstad A. (1993). Oral Hygiene, Periodontal Conditions and Carious Lesions in Patients Treated with Dental Bridges. A 15-Year Clinical and Radiographic Follow-Up Study. J. Clin. Periodontol..

[76] Lang N., Kiel R., Anderhalden K. (1983). Clinical and Microbiological Effects of Subgingival Restorations with Overhanging or Clinically Perfect Margins. J. Clin. Periodontol..

[77] Valderhaug J., Birkeland J. (1976). Periodontal Conditions in Patients 5 Years Following Insertion of Fixed Prostheses. J. Oral Rehabil..

[78] Ingber J.S., Rose L.F., Coslet J.G. (1977). the “biologic width”: A concept in periodontics and restorative dentistry. Alpha Omegan..

[79] Al-Sowygh Z.H. (2019). Does Surgical Crown Lengthening Procedure Produce Stable Clinical Outcomes for Restorative Treatment? A Meta-Analysis. J. Prosthodont..

[80] Mavrogiannis M., Ellis J.S., Seymour R.A., Thomason J.M. (2006). the efficacy of three different surgical techniques in the management of drug-induced gingival overgrowth. J. Clin. Periodontol..

[81] Maynard J.G., Wilson R.D. (1979). Physiologic dimensions of the periodontium significant to the restorative dentist. J. Periodontol..

[82] Pilalas I., Tsalikis L., Tatakis D.N. (2016). Pre-restorative crown lengthening surgery outcomes: A systematic review. J. Clin. Periodontol..

[83] Patil K., Khalighinejad N., El-Refai N., Williams K., Mickel A. (2019). the Effect of Crown Lengthening on the Outcome of Endodontically Treated Posterior Teeth: 10-year Survival Analysis. J. Endod..

[84] Ng Y.L., Mann V., Gulabivala K. (2011). a prospective study of the factors affecting outcomes of non-surgical root canal treatment: Part 2: Tooth survival. Int. Endod. J..

[85] Moghaddam A.S., Radafshar G., Taramsari M., Darabi F. (2014). Long-term survival rate of teeth receiving multidisciplinary endodontic, periodontal and prosthodontic treatments. J. Oral Rehabil..

[86] Lanning S.K., Waldrop T.C., Gunsolley J.C., Maynard J.G. (2003). Surgical crown lengthening: Evaluation of the biological width. J. Periodontol..

[87] Rocca G.T., Krejci I. (2007). Bonded indirect restorations for posterior teeth: The luting appointment. Quintessence Int..

[88] Rocca G.T., Krejci I. (2007). Bonded indirect restorations for posterior teeth: From cavity preparation to provisionalization. Quintessence Int..

[89] Castelo-Baz P., Argibay-Lorenzo O., Muñoz F., Martin-Biedma B., Darriba I.L., Miguéns-Vila R., Ramos-Barbosa I., López-Peña M., Blanco-Carrión J. (2021). Periodontal response to a tricalcium silicate material or resin composite placed in close contact to the supracrestal tissue attachment: A histomorphometric comparative study. Clin. Oral Investig..

[90] Stetler K.J., Bissada N.F. (1987). Significance of the width of keratinized gingiva on the periodontal status of teeth with submarginal restorations. J. Periodontol..

[91] Oppermann R.V., Gomes S.C., Cavagni J., Cayana E.G., Conceição E.N. (2016). Response to Proximal Restorations Placed Either Subgingivally or Following Crown Lengthening in Patients with No History of Periodontal Disease. Int. J. Periodontics Restor. Dent..

[92] Arora R., Narula S.C., Sharma R.K., Tewari S. (2013). Evaluation of supracrestal gingival tissue after surgical crown lengthening: A 6-month clinical study. J. Periodontol..

[93] Deas D.E., Moritz A.J., McDonnell H.T., Powell C.A., Mealey B.L. (2004). Osseous surgery for crown lengthening: A 6-month clinical study. J. Periodontol..

[94] Pontoriero R., Carnevale G. (2001). Surgical crown lengthening: A 12-month clinical wound healing study. J. Periodontol..

[95] Marzadori M., Stefanini M., Sangiorgi M., Mounssif I., Monaco C., Zucchelli G. (2018). Crown lengthening and restorative procedures in the esthetic zone. Periodontol. 2000.

[96] Ingber J.S. (1974). Forced eruption I. A method of treating isolated one and two wall infrabony osseous defects-rationale and case report. J. Periodontol..

[97] Sabri R. (1989). Principes et techniques [Crown lengthening by orthodontic extrusion. Principles and technics]. J. Parodontol..

[98] Minsk L. (2000). Orthodontic tooth extrusion as an adjunct to periodontal therapy. Compend. Contin. Educ. Dent..

[99] Oesterle L.J., Wood L.W. (1991). Raising the root. a look at orthodontic extrusion. J. Am. Dent. Assoc..

[100] Nobre C.M., de Barros Pascoal A.L., Albuquerque Souza E., Machion Shaddox L., Dos Santos Calderon P., de Aquino Martins A.R., de Vasconcelos Gurgel B.C. (2017). a systematic review and meta-analysis on the effects of crown lengthening on adjacent and non-adjacent sites. Clin. Oral Investig..

[101] Faria L.P., Almeida M.M., Amaral M.F., Pellizzer E.P., Okamoto R., Mendonça M.R. (2015). Orthodontic Extrusion as Treatment Option for Crown-Root Fracture: Literature Review with Systematic Criteria. J. Contemp. Dent. Pract..

[102] Buskin R., Castellon P., Hochstedler J.L. (2000). Orthodontic extrusion and orthodontic extraction in preprosthetic treatment using implant therapy. Pract. Periodontol. Aesthet. Dent..

[103] Brown I.S. (1973). the effect of orthodontic therapy on certain types of periodontal defects. I. Clinical findings. J. Periodontol..

[104] Bach N., Baylard J.F., Voyer R. (2004). Orthodontic extrusion: Periodontal considerations and applications. J. Can. Dent. Assoc..

[105] Smidt A., Gleitman J., Dekel M.S. (2009). Forced eruption of a solitary nonrestorable tooth using mini-implants as anchorage: Rationale and technique. Int. J. Prosthodont..

[106] Barone A., Derchi G., Rossi A., Marconcini S., Covani U. (2008). Longitudinal clinical evaluation of bonded composite inlays: A 3-year study. Quintessence Int..

[107] Dukic W., Dukic O.L., Milardovic S., Delija B. (2010). Clinical evaluation of indirect composite restorations at baseline and 36 months after placement. Oper. Dent..

[108] Huth K.C., Chen H.Y., Mehl A., Hickel R., Manhart J. (2011). Clinical study of indirect composite resin inlays in posterior stress-bearing cavities placed by dental students: Results after 4 years. J. Dent..

[109] Manhart J., Chen H.Y., Neuerer P., Scheibenbogen-Fuchsbrunner A., Hickel R. (2001). Three-year clinical evaluation of composite and ceramic inlays. Am. J. Dent..

[110] Thordrup M., Isidor F., Hörsted-Bindslev P. (2006). a prospective clinical study of indirect and direct composite and ceramic inlays: Ten-year results. Quintessence Int..

[111] Leirskar J., Nordbø H., Thoresen N.R., Henaug T., von der Fehr F.R. (2003). a four to six years follow-up of indirect resin composite inlays/onlays. Acta Odontol. Scand..

[112] Li H., Burrow M.F., Tyas M.J. (2000). Nanoleakage patterns of four dentin bonding systems. Dent. Mater..

[113] Nedeljkovic I., De Munck J., Vanloy A., Declerck D., Lambrechts P., Peumans M., Teughels W., Van Meerbeek B., Van Landuyt K.L. (2020). Secondary caries: Prevalence, characteristics, and approach. Clin. Oral. Investig..

[114] Flores-de-Jacoby L., Zafiropoulos G.G., Ciancio S. (1989). Effect of crown margin location on plaque and periodontal health. Int. J. Periodontics Restorative Dent..

[115] Kemp-Scholte C.M., Davidson C.L. (1990). Marginal integrity related to bond strength and strain capacity of composite resin restorative systems. J. Prosthet. Dent..

[116] Cavalcanti A.N., Mitsui F.H., Ambrosano G.M., Marchi G.M. (2007). Influence of adhesive systems and flowable composite lining on bond strength of class II restorations submitted to thermal and mechanical stresses. J. Biomed. Mater. Res. B Appl. Biomater..

[117] Castañeda-Espinosa J.C., Pereira R.A., Cavalcanti A.P., Mondelli R.F. (2007). Transmission of composite polymerization contraction force through a flowable composite and a resin-modified glass ionomer cement. J. Appl. Oral Sci..

[118] Litonjua L.A., Andreana S., Bush P.J., Tobias T.S., Cohen R.E. (2003). Noncarious cervical lesions and abfractions: A re-evaluation. J. Am. Dent. Assoc..

[119] Agarwal R.S., Hiremath H., Agarwal J., Garg A. (2015). Evaluation of cervical marginal and internal adaptation using newer bulk fill composites: An in vitro study. J. Conserv. Dent..

[120] Francis A.V., Braxton A.D., Ahmad W., Tantbirojn D., Simon J.F., Versluis A. (2015). Cuspal Flexure and Extent of Cure of a Bulk-fill Flowable Base Composite. Oper. Dent..

[121] Moorthy A., Hogg C.H., Dowling A.H., Grufferty B.F., Benetti A.R., Fleming G.J. (2012). Cuspal deflection and microleakage in premolar teeth restored with bulk-fill flowable resin-based composite base materials. J. Dent..

[122] Kamath U., Sheth H., Vigneshwar *!!! REPLACE !!!* (2012). Role of delayed light polymerization of a dual-cured composite base on marginal adaptation of class II posterior composite open-sandwich restoration. Indian J. Dent. Res..

[123] Rodrigues Junior S.A., Pin L.F., Machado G., Della Bona A., Demarco F.F. (2010). Influence of different restorative techniques on marginal seal of class II composite restorations. J. Appl. Oral Sci..

[124] Lin C.L., Chang Y.H., Chang C.Y., Pai C.A., Huang S.F. (2010). Finite element and Weibull analyses to estimate failure risks in the ceramic endocrown and classical crown for endodontically treated maxillary premolar. Eur. J. Oral Sci..

[125] Eraslan Ö., Eraslan O., Eskitaşcıoğlu G., Belli S. (2011). Conservative restoration of severely damaged endodontically treated premolar teeth: A FEM study. Clin. Oral Investig..

[126] Dietschi D., Duc O., Krejci I., Sadan A. (2007). Biomechanical considerations for the restoration of endodontically treated teeth: A systematic review of the literature—Part 1. Composition and micro- and macrostructure alterations. Quintessence Int..

[127] ElAyouti A., Serry M.I., Geis-Gerstorfer J., Löst C. (2011). Influence of cusp coverage on the fracture resistance of premolars with endodontic access cavities. Int. Endod. J..

[128] Dietschi D., Duc O., Krejci I., Sadan A. (2008). Biomechanical considerations for the restoration of endodontically treated teeth: A systematic review of the literature, Part II (Evaluation of fatigue behavior, interfaces, and in vivo studies). Quintessence Int..

